# Ectopic expression of aPKC-mediated phosphorylation in p300 modulates hippocampal neurogenesis, CREB binding and fear memory differently with age

**DOI:** 10.1038/s41598-018-31657-2

**Published:** 2018-09-10

**Authors:** Charvi Syal, Matthew Seegobin, Sailendra Nath Sarma, Ayden Gouveia, Karolynn Hsu, Yosuke Niibori, Ling He, Fredric E. Wondisford, Paul W. Frankland, Jing Wang

**Affiliations:** 1 Regenerative Medicine Program, Ottawa Hospital Research Institute, Ottawa, K1H 8L6 Canada; 2 Department of Cellular and Molecular Medicine, University of Ottawa, Ottawa, K1H 8M5 Canada; 3 Brain and Mind Research Institute, University of Ottawa, Ottawa, K1H 8M5 Canada; 4 Neurosciences and Mental Health, Hospital for Sick Children, Toronto, ON M5G 1X8 Canada; 5 Department of Pediatrics and Medicine, Johns Hopkins Medical School, Baltimore, MD 21287 USA; 6 Department of Medicine, Rutgers-Robert Wood Johnson Medical School, New Brunswick, NJ 08901 USA; 7 Department of Psychology, University of Toronto, Toronto, M5G 1X5 Canada; 8 Department of Physiology, University of Toronto, Toronto, M5G 1X5 Canada

## Abstract

Epigenetic modifications have become an emerging interface that links extrinsic signals to alterations of gene expression that determine cell identity and function. However, direct signaling that regulates epigenetic modifications is unknown. Our previous work demonstrated that phosphorylation of CBP at Ser 436 by atypical protein kinase C (aPKC) regulates age-dependent hippocampal neurogenesis and memory. p300, a close family member of CBP, lacks the aPKC-mediated phosphorylation found in CBP. Here, we use a phosphorylation-competent *p300* (G442S) knock-in (KI) mouse model that ectopically expresses p300 phosphorylation in a homologous site to CBP Ser436, and assess its roles in modulating hippocampal neurogenesis, CREB binding ability, and fear memory. Young adult (3 months) *p300*G422S-KI mice exhibit enhanced hippocampal neurogenesis due to increased cell survival of newly-generated neurons, without alterations in CREB binding and contextual fear memory. On the other hand, mature adult (6 months) *p300*G422S-KI mice display reduced CREB binding, associated with impaired contextual fear memory without alterations in hippocampal neurogenesis. Additionally, we show that repulsive interaction between pS133-CREB and pS422-p300G422S may contribute to the reduced CREB binding to *p300*G422S. Together, these data suggest that a single phosphorylation change in p300 has the capability to modulate hippocampal neurogenesis, CREB binding, and associative fear memory.

## Introduction

Increasing evidence shows that hippocampal memory plasticity is not only regulated by synapse-specific modification^1^, but also modulated by adult neurogenesis process^2,3^. Long-lasting changes in the strength of hippocampal neuron synaptic connections contribute to hippocampal memory formation and storage^1^. On the other hand, adult-born neurons continuously produced from the hippocampal subgranular zone (SGZ)^2,4,5^ are crucial for hippocampal neuronal addition, promoting new memory formation during adulthood^5–9^. Adult neural stem and progenitor cells (NPCs) in the SGZ mainly produce transit-amplifying cells/neuroblasts, which give rise to granule neurons in the hippocampal dentate gyrus^2^. Interestingly, both synaptic plasticity and adult hippocampal neurogenesis share a common molecular substrate, CREB, a key transcription factor in regulating cell excitability, synaptic plasticity, neurogenesis, cell survival, and memory formation^10–15^.

When Ser133 in CREB is phosphorylated, it recruits CREB binding protein (CBP)/p300, a family of histone acetyltransferases, and promotes CREB-mediated gene transcription^16,17^. We recently identified that the aPKC-mediated CBP phosphorylation at Ser436 acts as compensatory signaling to maintain hippocampal neurogenesis and hippocampal dependent memory during the aging process^18^. The Ser 436 phosphorylation in CBP retains the interaction between CBP and CREB in mature adult mice (6 months) where CREB S133 phosphorylation is significantly reduced^18^. Interestingly, p300, a close family member of CBP, does not have the aPKC-mediated phosphorylation site^19^. Previous study has shown that this single phosphorylation difference between CBP and p300 in liver cells allows them to have distinct roles in regulating blood glucose levels on the basis of their different CREB binding abilities^19,20^. In this regard, it is important to use a phosphorylation-competent *p300*G422S knock-in mouse model, creating an extra aPKC-phosphorylation site in p300, to decipher underlying mechanisms through which the single aPKC-mediated phosphorylation regulates CREB binding, hippocampal memory and adult neurogenesis.

Many studies have shown that both CBP and p300 are involved in contextual fear memory formation via their histone acetyltransferase (HAT) activity^21–25^. In addition, histone deacetylase inhibitors enhance fear memory processes by the activation of CREB-mediated expression of memory-associated genes, such as NR4A and DUSP^25–27^. Although the functional role of p300/CBP in contextual fear memory formation is well studied, the direct signal that controls p300/CBP function and its underlying mechanisms in fine-tuning hippocampal fear memory remains unknown.

Our findings show that ectopic expression of aPKC-mediated phosphorylation using a phosphorylation-competent *p300*G422S KI mouse model is sufficient to increase adult hippocampal neurogenesis by reducing cell death of newborn neurons in 3-month-old *p300*G422S-KI mice without altering CREB binding and fear memory. In contrast, the 6-month-old *p300*G422S-KI mice display reduced CREB binding and subsequently decreased CREB-mediated gene expression, associated with impaired hippocampal contextual fear memory in the absence of changes in adult neurogenesis. We further show that repulsive interaction between pS133-CREB and pS422-*p300*G422S contributes to the reduced CREB binding in *p300*G422S at the age of 6 months. Together, these data suggest that a single phosphorylation change in p300 has the capability to modulate hippocampal neurogenesis, CREB binding, and contextual fear memory differently by aging.

## Results

### *p300*G422S-KI increases adult neurogenesis at the age of 3 months by reducing cell death of newborn neurons

**Figure 1:**
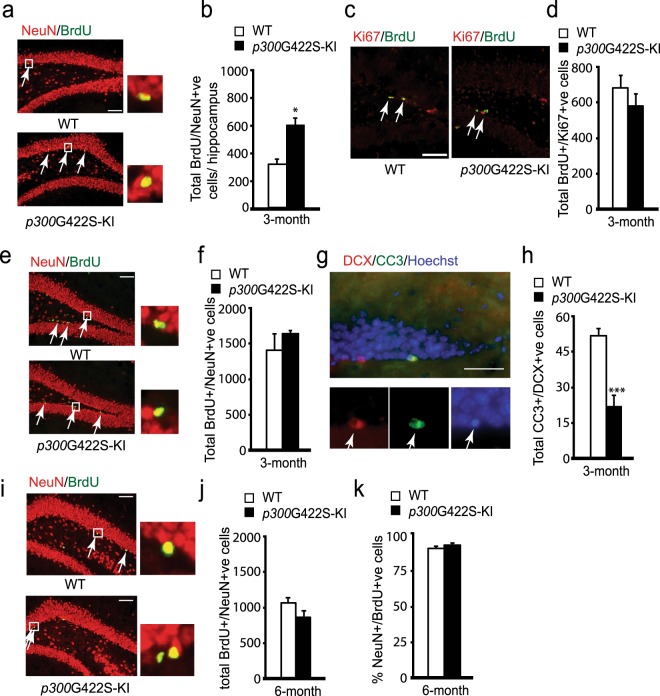
*p300*G422S-KI increases adult neurogenesis at the age of 3 months by reducing cell death of newborn neurons. (**a**) Representative images of hippocampal sections from 3-month *p300*G422S-KI (homologous *p300*G422S-KI) and their WT, sacrificed 30 days after BrdU injections, and stained for BrdU (green) and NeuN (red). Arrows represent BrdU+/NeuN+ neurons. Scale bar = 20 µm. (**b**) Quantitative analysis of the total number of BrdU+/NeuN+ newborn neurons in the hippocampi from 3 months WT and *p300*G422S-KI, as shown in (**a**). (**c**) Representative fluorescence images of hippocampal sections from 3 months WT and *p300*G422S-KI mice, sacrificed one day after BrdU injections, stained for BrdU (green) and Ki67 (red). Arrows denote BrdU+/Ki67+ proliferating cells. Scale bar = 20 μm. (**d**) Quantitative analysis of the total number of BrdU+/Ki67+ proliferating cells in the hippocampi from 3 months *p300*G422S-KI and their WT littermates. (**e**) Representative images of hippocampal sections from 3 months WT and *p300*G422S-KI mice, sacrificed 12 days after BrdU injections, stained for BrdU (green) and NeuN (red). Arrows represent BrdU+/NeuN+ neurons. Scale bar = 20 µm. (**f**) Quantitative analysis of the total number of BrdU+/NeuN+ neurons in the hippocampi from WT and *p300*G422S-KI mice (3-month) as shown in (**e**). (**g**) Representative images of hippocampal sections from 3-month WT mice, stained for cleaved caspase 3 (CC3) (green) and DCX (red). Arrows denote CC3+/DCX+ cells. Scale bar = 10 μm. (**h**) Quantitative analysis of the total number of CC3+/DCX+ cells in the hippocampi from 3 months *p300*G422S-KI and their WT littermates. (**i**) Representative images of hippocampal sections from 6 months WT and *p300*G422S-KI mice, sacrificed 12 days after BrdU injections, stained for BrdU (green) and NeuN (red). Arrows represent BrdU+/NeuN+ neurons. Scale bar = 20 µm. (**j**,**k**) Quantitative analysis of total number (**j**) and the proportion (**k**) of BrdU+/NeuN+ cells in the hippocampi from WT and *p300*G422S-KI mice (6 months) as shown in (**i**). The boxed areas were shown at higher magnification on the right panels in (a,e,i). *p < 0.05; ***p < 0.001, n = 4 animals for each group.

To ask whether ectopic expression of aPKC-mediated phosphorylation in p300 had an impact on adult hippocampal neurogenesis, we first performed BrdU *in vivo* labeling experiments in 3- month-old *p300*G422S-KI mice. BrdU (60 mg/kg; i.p.) was injected four times at three-hour interval to the mice, which were sacrificed 30 days later. We performed immunohistochemistry for BrdU and the mature neuron marker, NeuN, on hippocampal brain sections. A significant increase in the total number of BrdU^+^/NeuN^+^ newborn neurons in *p300*G422S-KI mice was observed (Fig. 1a,b).

To understand underlying cellular mechanisms that contribute to increased neurogenesis in the *p300*G422S-KI mice, BrdU *in vivo* experiments were first performed at two early time points, 1 and 12 days post BrdU injections. At 1 day after BrdU injection, the number of proliferating NPCs that express both BrdU and Ki67 was not changed between wild type (WT) and *p300*G422S-KI mice (Fig. 1c,d). At 12 days after BrdU injections, the number of co-labelled BrdU+/NeuN+ neurons was unaltered in *p300*G422S-KI mice as well (Fig. 1e,f). Interestingly, when brain sections were immunostained with an apoptotic marker, cleaved caspase-3 (CC3), and a marker for neuroblasts/newborn neurons, doublecortin (DCX) as we have done previously^18^, we showed that most of CC3-positive cells (~80%) were DCX-positive neuroblasts/newborn neurons. Importantly, the number of double-labelled DCX+/CC3+ cells was decreased in *p300*G422S-KI mice (Fig. 1g,h), and each CC3+ cell contained a condensed apoptotic nucleus (Fig. 1g). Together, these results show that 3-month-old *p300*G422S-KI mice display increased cell survival of newborn neurons, ultimately leading to enhanced neurogenesis at the age of 3 months. These phenotypes were totally opposite to those previously identified in 3-month phosphomutant *CBP*S436A-KI mice showing reduced neurogenesis due to increased cell death of newborn neurons^18^.

### *p300*G422S-KI impairs CREB binding but does not change adult neurogenesis at the age of 6 months

**Figure 2:**
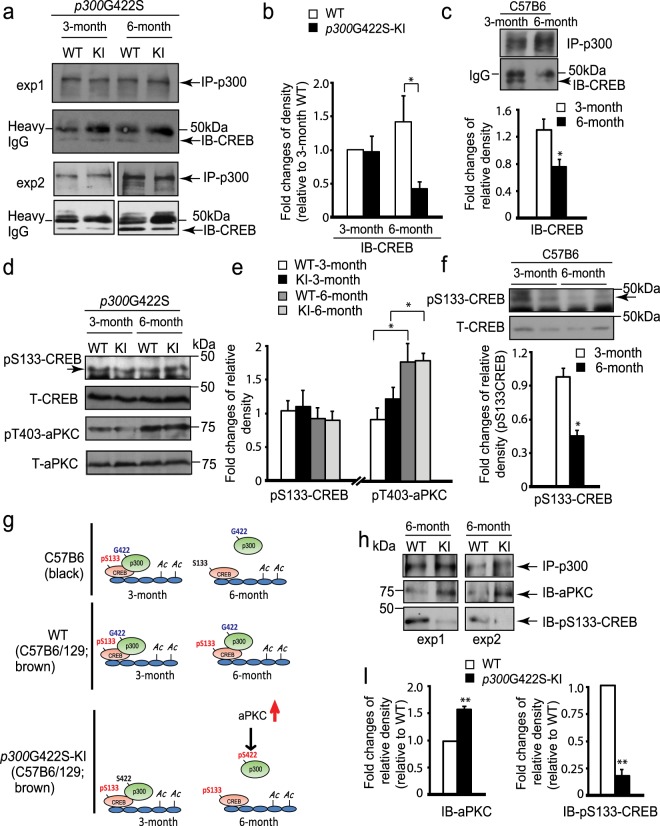
*p300*G422S-KI shows the impaired CREB binding to p300 at the age of 6 but not 3 months. (**a**) Co-immunoprecipitation analysis of the interaction between p300 and CREB in the hippocampi of WT and *p300*G422S-KI mice at 3 and 6 months. Hippocampal lysates were immunoprecipitated with a p300 antibody, washed and blotted with the indicated antibody. Arrow indicates CREB expression band. (**b**) The graph indicates the fold changes of the relative pulled-down CREB protein over the total p300 amounts, as determined by densitometry and normalized to samples from 3 months WT, (two-way ANOVA: F (1,16) = 4.86, p = 0.04; Tukey’s multiple comparisons, *p < 0.05, n = 5). (**c**) Co-immunoprecipitation analysis of the interaction between p300 and CREB in C57B6 strain hippocampi at the age of 3 and 6 months as described in (**a**). The graph data was normalized to one of 3 months old C57B6 samples. (**d**) Western blot analysis for pS133-CREB and pT410/403-aPKC zeta/iota in hippocampal extracts from 3 and 6 months WT and *p300*G422S-KI mice. Blots were reprobed for total CREB or aPKC as loading controls. (**e**) Graphs show relative levels of pS133-CREB and pT410/403-aPKC over total CREB and aPKC, respectively, normalized to one of 3 months WT samples. (**f**) Western blot analysis for pS133-CREB in hippocampal extracts from 3 and 6 months C57B6 mouse strain. Blots were reprobed for total CREB as a loading control. Graphs show relative levels of pS133-CREB over total CREB, normalized to one of 3 months C57B6 samples. (**g**) Schematic model showing that both pS133-CREB and pS422-p300 determine the interaction between p300 and CREB. (**h**) Co-immunoprecipitation analysis of the association of p300 with pS133-CREB and total aPKC in the hippocampi of 6 months WT and *p300*G422S-KI mice. (**i**) The graph indicates the fold changes of the relative pulled-down pS133CREB protein and total aPKC over the total p300 amounts, as determined by densitometry and normalized to 6 months WT samples. exp1: individual experiment 1; exp2: individual experiment 2. *p < 0.05; **p < 0.01, n = 3–5 animals for each group. Images derived from different part of the same gel were cropped for layout reasons. Full-length blots/gels are presented in Supplementary Fig. 2.

Since our previous study showed impaired hippocampal neuronal differentiation in 6-month-old phosphomutant *CBP*S436A-KI mice^18^, we examined hippocampal neuronal differentiation in 6 months phosphorylation-competent *p300*G422S-KI mice. We found no difference in terms of number and percentage of newborn neurons produced from the SGZ NPCs between WT and *p300*G422S-KI mice (Fig. 1i,k), labeled by BrdU as described previously. On the other hand, we observed the reduced CREB binding to p300 in 6-month *p300*G422S-KI hippocampal tissues (Fig. 2a,b) compared to their WT littermates. More interestingly, when mice grow from 3 to 6 months, WT littermates (a mixed C57B6/129 strain, brown color) for *p300*G422S-KI showed no significant changes for the interaction between CREB and p300 (Fig. 2a,b), while another independent C57B6 strain (black color) showed reduced CREB binding to p300 (Fig. 2c). To further decipher underlying mechanisms that regulate the interaction between CREB and p300 in both strains of mice, we performed western blot analysis to assess CREB phosphorylation at S133 (pS133-CREB), which is a key regulator for the association of CREB with p300. We found that pS133-CREB levels were significantly reduced in C57B6 strain (black color) mice from 3 to 6 months, but remained unchanged in both *p300*G422S-KI and their WT littermates (mixed C57B6/129 strain, brown color) (Fig. 2d–f). We also showed that pS133-CREB levels were reduced in C57B6 strain (black color) as compared to the p300G422S line (a mixed C57B6/129 strain, brown color) at the age of 6 months (Supplementary Fig. 1). In addition, we measured aPKC activity, illustrated by aPKC threonine phosphorylation at T410/403. Western blot analysis showed that both *p300*G422S-KI and their WT littermates exhibited a significant increase in pT410/403-aPKC from 3 to 6 months hippocampal extracts (Fig. 2d,e). In this regard, we propose the model that the co-existence of both pS133-CREB and pS422-p300 dissociates the interaction between p300 and CREB, while pS133-CREB alone enhances the association between p300 and CREB (Fig. 2g). To support the model, we performed co-immunoprecipitation (co-IP) experiments using anti-p300 to pull-down both WT p300 and p300G422S from 6 months hippocampal extracts. We showed that p300G422S formed a strong binding to aPKC compared to WT p300, indicating the phosphorylated status of p300 at S422 (pS422-p300). In contrast, the reduced binding of pS133-CREB to p300G422S was observed in the same IP complex (Fig. 2h,i). Thus, the ectopic expression of aPKC-mediated phosphorylation in *p300*G422S-KI repulses the pS133-CREB at the age of 6 months, leading to the dissociation between p300 and CREB (Fig. 2h,i).

**Figure 3:**
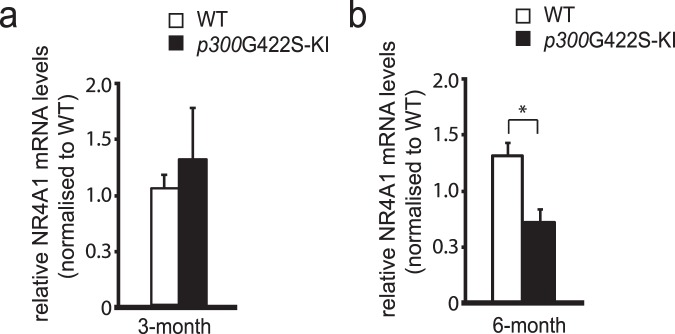
*p300*G422S-KI reduces CREB-mediated gene expression at the age of 6 month. 6 months but not 3 months *p300*G422S-KI hippocampi have reduced expression of a CREB-mediated gene, *NR4A1*. *p < 0.05. n = 4–6 animals for each group.

To further assess whether the dissociation between p300 and CREB in 6 months *p300*G422S-KI hippocampi led to the reduction of CREB-mediated gene expression, which is known to be involved in hippocampal fear memory formation. We performed RT-PCR analysis using both WT and *p300*G422S hippocampal tissues and observed that a CREB-mediated gene, NR4A1, was reduced in 6-month-old *p300*G422S-KI as compared to their WT littermates but not in 3 months *p300*G422S-KI (Fig. 3a,b).

### *p300*G422S-KI modulates hippocampal contextual fear memory at the age of 6 months

**Figure 4:**
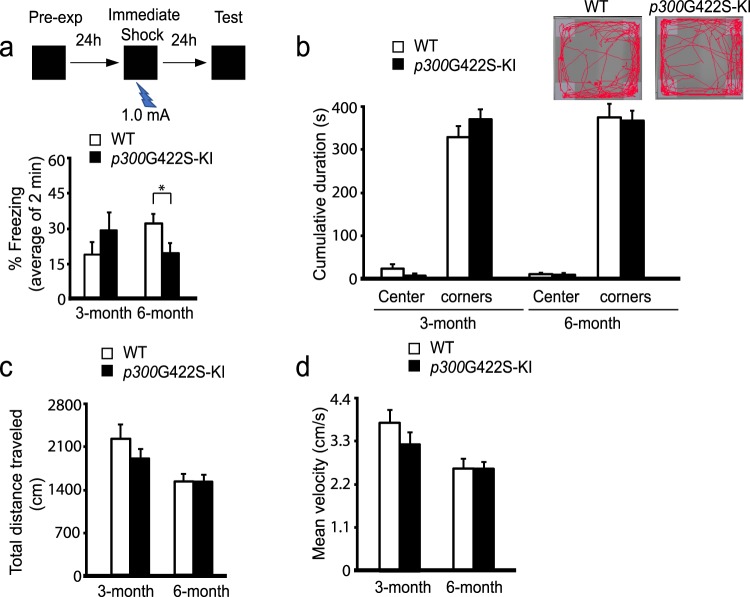
*p300*G422S-KI at the age of 6 months shows reduced hippocampal contextual fear memory. (**a**) Both 3 and 6 months homozygous *p300*G422S-KI and their WT littermates, were pre-exposed to the conditioning context at day one, and received an immediate shock (1.0 mA, 2 seconds) within the same context at day two. Percentage of time spent freezing within the first two minutes when the mice were re-placed in the conditioning context at day three without shock. *p < 0.05 (**b**) Open field test was performed in an open box for 10 minutes. The cumulative time spent within the center and all 4 corners of the box was analyzed. Insets show representative traces of WT and *p300*G422S -KI mouse during the course of the open field test. (**c**) Analysis of the total distance travelled during the open field test for WT and *p300*G422S -KI mice at the age of 3 and 6 months. (**d**) Analysis of the mean velocity during the open field test at the age of 3 and 6 months. n = 8–13 animals for each group.

To assess hippocampal fear memory, we used a context pre-exposure task, a version of contextual fear memory that is critically dependent on the hippocampus^18,28,29^. In this task, the mice were exposed to the context alone for 24 hours, followed by the foot shock associated with the experienced context. This strategy temporally separated the context acquisition phase from the association of the context with the shock (Fig. 4a). 6-month-old homozygous *p300*G422S-KI mice, but not 3-month-old, exhibited the decreased percentage of time spent in a freezing position, suggesting reduced associative fear memory (Fig. 4a). To rule out the possibilities that the contextual fear memory alterations are due to nonspecific impairments in motor function or anxiety, we assessed mean velocity, distance traveled, and anxiety behaviors in the open field test (Fig. 4b–d) and observed no changes between WT and homozygous *p300*G422S-KI mice.

Together, our results show that ectopic expression of aPKC-mediated phosphorylation in *p300*G422S, the homologous site of CBP at Ser436, modulates hippocampal contextual fear memory at the age of 6 months, possibly via CREB binding-dependent pathway.

## Discussion

Our present data demonstrate that ectopic expression of aPKC-mediated phosphorylation in p300, which normally lacks the phosphorylation site, modulates hippocampal neurogenesis, CREB binding and hippocampal contextual fear memory differently by age. In 3-month-old mice, the phosphorylation-competent *p300*G422S-KI mice show enhanced adult neurogenesis due to reduced cell death of newborn neurons, without alterations in CREB binding and hippocampal contextual fear memory. Oppositely, 6-month-old *p300*G422S-KI mice display impaired fear memory in the absence of changes in hippocampal neurogenesis, most likely due to reduced CREB binding and decreased expression of CREB-mediated genes such as NR4A1, which play an important role in hippocampal fear memory formation.

Since p300 does not have the aPKC-mediated phosphorylation site that is found in CBP at Ser436, the phosphorylation-competent *p300*G422S-KI mouse is considered as a gain of function model for the aPKC-CBP pathway^19^. Interestingly, the increased neurogenesis that is due to enhanced cell survival of newborn neurons in 3-month *p300*G422S-KI mice is opposite to the phenotype previously observed in 3-month phosphomutant *CBP*S436A-KI mice, which show reduced neurogenesis as a result of increased cell death of newborn neurons. Importantly, CREB binding ability to CBP/p300 is not altered in both young adult phosphorylation-competent (*p300*G422S-KI) and phosphomutant *CBP*S436A-KI mice^18^. These findings suggest that the aPKC-mediated phosphorylation in p300/CBP modulates hippocampal neurogenesis through regulating cell death of adult-born neurons independent of CREB binding. p53, a non-histone substrate of CBP/p300, is one candidate that is considered to play a key role in mediating the cell death effect regulated by the aPKC-CBP/p300 pathway. CBP/p300-mediated p53 acetylation has been shown to increase p53 protein stability and enhance the binding of p53 to apoptotic gene promoters, such as BAX and PUMA^30^. The conformation changes in CBP/p300 due to phosphorylation KI mutations possibly modulate its binding and acetylating ability to p53, consequently leading to changes in cell death rate of newborn neurons. Future work will focus on deciphering the p53 mechanisms in mediating the aPKC-CBP/p300 pathway in regulating newborn cell survival.

To our surprise, *p300*G422S-KI mice showed the same impaired CREB binding ability as *CBP*S436A-KI mice in mature adult (6 months). How did this happen? We discovered that the aPKC-mediated pS436-CBP/pS422-p300 has the same capability as pS133-CREB to regulate the association of CREB with CBP/p300. In the mature adult (6 months) *CBP*S436A mouse strain, pS133-CREB is significantly reduced compared to that in 3 months, thus aPKC-mediated pS436 in CBP acts as a compensatory mechanism to maintain the association between CBP and CREB in 6 months hippocampi^18^. In this regard, 6 months *CBP*S436A-KI hippocampi lack both pS133-CREB and pS436-CBP, therefore showing impaired binding ability to CREB^18^. Conversely, pS133-CREB sustains from 3 to 6 months in *p300*G422S mouse strain (a mixed C57B6/129 strain, brown color) regardless of genotypes (Fig. 2d,e). The impaired CREB binding to *p300*G422S-KI in 6 months hippocampi reveals that simultaneous pS133-CREB and pS422-p300 creates a repulsive interaction between CREB and p300. This is consistent with the previously published study in hepatic tissue^19^. In summary, the aPKC-mediated pS436-CBP/pS422-p300G422S and pS133-CREB, individually promotes the association between CBP/p300 and CREB, while co-appearance of both phosphorylation on CBP/p300 and CREB in the cells disrupts the association between CBP/p300 and CREB (Fig. 2g).

Interestingly, the reduced CREB binding to p300G422S did not impair hippocampal neuronal differentiation and maturation at the age of 6 months, suggesting that p300 is not important in regulating neuronal differentiation and maturation as CBP^18^. In contrast, CREB-mediated gene expression (NR4A) that is essential for fear memory formation are significantly reduced in *p300*G422S-KI mice, implying that p300 instead plays an essential role in maintaining CREB-modulated expression of memory-associated genes. Recently, NR4A, a nuclear receptor family, has been discovered to be transcriptionally activated by extrinsic signals such as HDAC inhibitor or PKA-CREB signaling cascade, consequently leading to increased memory-associated gene expression^26^. Our data supports the concept that the recruitment of p300 to CREB is required for forming hippocampal contextual fear memory by promoting NR4A gene expression.

Altogether, we show here that phosphorylation-competent young adult (3 months) *p300*G422S mice display enhanced hippocampal neurogenesis due to reduced cell death of newborn neurons. Conversely, mature adult (6 months) *p300*G422S mice show impaired hippocampal contextual fear memory, which is most likely due to reduced CREB binding and subsequently decreased CREB-mediated gene expression that is important for fear memory formation.

## Methods

### Animals and drug treatment

All animal use was approved by the Animal Care Committees of the Hospital for Sick children and the University of Ottawa on the basis of the Canadian Council of Animal Care policies. *p300G422S* mice (a mixed C57B6/129 strain)^19^ and wild type C57B6 mice (Charles River Laboratories) were maintained on a 12 h light/12 h dark cycle with *ad libitum* access to food and water.

### BrdU *in vivo* labeling

BrdU *in vivo* labelling was performed as described before^18^. Briefly, in one set of experiments, we injected mice with BrdU (Sigma-Aldrich, intraperitoneally (i.p.), 100 mg/kg) once and then mice were sacrificed 24 hours later. In a second set of experiments, we injected mice with BrdU (i.p. 100 mg/kg) once daily for 3 days. The mice were then sacrificed 9 days after the last BrdU injection. In a third set of experiments, we injected mice with BrdU (i.p. 60 mg/kg) 4 times at 3-hour intervals. mice were sacrificed 30 days later.

Following the three sets of BrdU-chasing experiments, the mice were sacrificed by a lethal dose of pentobarbital and perfused transcardially with PBS and 4% paraformaldehyde. Brains were post-fixed, cryoprotected and cryosectioned at 20 μm for hippocampus. Every tenth hippocampal section was analyzed immunohistochemically for BrdU, NeuN, Ki67 as previously described^18^.

### Immunohistochemistry, microscopy, and quantification

Immunohistochemistry of brain sections was performed as previously described^18^. Sections were post-fixed with 4% PFA, blocked and permeabilized with 10% normal goat serum and 0.3% Triton-X, and then sections were incubated with primary antibodies at 4 °C overnight, with secondary antibodies at room temperature for 1 hour, counterstained with Hoechst 33343 (1:2000, Sigma-Aldrich) and mounted using Permafluor (Thermo Fisher Scientific). For BrdU colabelling with NeuN, or Ki67, sections were incubated in 1 N HCl at 60 °C for 30 min, rinsed in PBS, incubated in rat anti-BrdU antibody at 4 °C overnight, in Alexa 488 donkey anti-rat antibody for 1 hour and then sequentially immunostained with anti-NeuN and anti-Ki67 followed by Alexa Fluor-conjugated secondary antibodies.

Digital image acquisition was performed using either a Zeiss Axioplan 2 fluorescent microscopy with Zeiss Axiovision software that contains z-axis capability, or a Zeiss LSM 510 confocal microscopy using Zeiss Zen Pro software V2.0 (Oberkochen, Germany). 10–15 images were captured in the Z-axis per section at a maximum of 1um apart and processed as an optical stack of 10–15 scanned slices for quantification.

For quantification, positive cells were quantified using a modified stereological method that have been extensively used^31–36^. We exhaustively quantified every positive cell within dentate gyrus region including SGZ, GCL and hilus. Thus we used an area sampling fraction of 1 as is commonly utilized for counting rare populations of cells^37,38^ since the raw counts for the numbers of positive cells were low according to disector/fractionator standards^36,39–41^ and the cells are not evenly distributed within the dentate gyrus. Given recent work suggesting the absence of lost caps in perfusion fixed tissue and potential bias that can occur with traditional use of guard zones^42–44^, we did not employ a guard zone and used the same method to quantify both wild type and knock-in mice brain sections. We sampled 1 in every 10 sections throughout the septotemporal axis of the hippocampal structure (−1.3 mm to −3.70 mm relative to bregma referring to the rostral-caudal coordinates) by an examiner that was blind to group assignments. Since the section sampling fraction was 1/10, the resulting raw count for each region was multiplied by 10 to obtain an estimate of cell numbers per dentate gyrus.

### Co-immunoprecipitation

Isolated hippocampal tissues were homogenized and lysed, as described previously^18^, in lysis buffer (25 mM Tris, pH = 7.4, 10 mM NaCl, 2 mM EDTA, 1 mM EGTA, 0.5% Triton-100, 10% glycerol) containing 1 mM PMSF, 1 mM sodium orthovanadate, 20 mM sodium fluoride, 10 μg/ml aprotinin and 10 μg/ml leupeptin. The extractions were sonicated 3 times with 5 seconds pulses at 1 minute intervals. Then, 1 mg protein lysate from each sample was incubated with 35ul protein A conjugated magnetic beads and 3 ug anti-p300 antibody or normal rabbit IgG antibody at 4 °C overnight. Following that, the magnetic beads were rinsed 3 times with lysis buffer, boiled with sample buffer, and loaded on a 8–15% gradient SDS-PAGE gel.

### Western blot analysis and densitometry

Hippocampal tissues were lysed in lysis buffer (25 mM Tris, pH = 7.4, 10 mM NaCl, 2 mM EDTA, 1 mM EGTA 0.5% Triton-100, 10% glycerol) containing 1 mM PMSF, 1 mM sodium orthovanadate, 20 mM sodium fluoride, 10 μg/ml aprotinin and 10 μg/ml leupeptin. 100 μg protein lysates were resolved on a 10% SDS-PAGE gel, and western blots performed as previously described^18^. Densitometry was performed using Image J.

### Context pre-exposure fear conditioning

The pre-exposure context fear memory was performed as described previously^18^. Briefly, each mouse was placed in the conditioning context chamber for 10 min. No shocks were delivered in this phase of the experiment. Twenty-four hours later, each mouse was placed in the conditioning context and, 5 s later, received a foot shock (2 s, 1.0 mA). Twenty-four hours after immediate shock training, each mouse was placed in the conditioning context and freezing was assessed for a 2-min period. During this period, no shock was presented.

### Open field test

Open field test was done at the University of Ottawa Behavior Core. Mice were individually placed in a 45 cm × 45 cm × 45 cm open field chamber for 10 min, and Ethovision software was used to record and analyze the distance traveled, locomotion speed, and amount of time the mice spent in respective zones (outer, middle, and center) of the box.

### RNA extraction, cDNA synthesis, and quantitative real-time polymerase chain reaction

Mice were sacrificed from their home cages for the evaluation of CREB-mediated gene expression changes. Whole hippocampi were dissected, and frozen in liquid nitrogen. RNA was extracted from hippocampal tissue using TRIzol plus purification kit (Ambion). cDNA was synthesized from RNA using a retroscript kit from Qiagen. Quantitative real-time polymerase chain reaction (qPCR) was carried out using the Sensifast^tm^ SYBR-green master mix (Bioline) and 400 nM primers (final concentration) on the Stratagene MX3000 using MXPro qPCR software. Cycling parameters were 95 °C for 10 min followed by 40 cycles of 95 °C (30 sec) and 60 °C (1 min), ending with a melting curve analysis to assess the amplification of a single amplicon. All reactions were performed in duplicate, with the median cycle time used for analysis. GAPDH was used as a housekeeping gene against all experimental genes. Primer sequences were as following: *NR4A1* forward 5′-AAAATCCCTGGCTTCATTGAG-3′, reverse 5′-TTTAGATCGGTATGCCAGGCG-3′.

### Antibodies

For immunohistochemistry, the primary antibodies used were rabbit anti-cleaved caspase 3 (1:1000; Cat#9661, Cell Signaling Technology, Beverly, MA), mouse anti-Ki67 (1:200; Cat#556003, BD Pharmingen, Heidelberg, Germany), rat anti-BrdU (1:200; Cat#OBT0030G, Accurate Chemical), mouse anti-NeuN (1:500; Cat#MAB377, Chemicon). The secondary antibodies used were Alexa Fluor 555-conjugated goat anti-mouse IgG (1:1000; Cat#A21422, Molecular Probes), Alexa Fluor 488-conjugated goat anti-rat IgG (1:1000; Cat#A21208, Molecular Probe), Alexa Fluor 488-conjugated goat anti-rabbit IgG (1:1000; Cat#4412, Cell Signaling), Alexa Fluor 555-conjugated donkey anti-goat IgG (1:1000; Cat#A21432, Molecular Probes), Alexa Fluor 647-conjugated goat anti-mouse IgG (1:1000; Cat#A21237, Molecular Probes). For western blots, the primary antibodies were, rabbit anti-p-aPKCζ/ι (T410/403)(1:500, Cat#9378, Cell Signaling), mouse anti- aPKCζ/ι (1:500; Cat#610175, BD), rabbit anti-p-CREB (S133)(1:500; Cat#9198, Cell Signaling), mouse anti- CREB (1:500; Cat#9104, Cell Signaling), rabbit anti-p300 (1:100; Cat#sc-585, Santa-Cruz), and mouse anti-p300 (1:1000, Cat#ab14984, Abcam). Secondary antibodies for western blots were HRP-conjugated goat anti-mouse or anti-rabbit (1:4000; Cat#7076 and #7074, Boehringer Mannheim).

### Statistics

A two-tailed Student’s t test was used for statistical analyses, unless otherwise indicated. Error bars indicate the standard error of the mean (SEM).

## Electronic supplementary material

Supplementary figures

## References

[1] Kandel ER (2009). The Biology of memory: a forty-year perspective. J. Neurosci..

[2] Goncalves JT, Schafer ST, Gage FH (2016). Adult neurogenesis in the hippocampus: from stem cells to behavior. Cell.

[3] Song J, Christian K, Ming G, Song H (2012). Modification of hippocampal circuitry by adult neurogenesis. Dev. Neurobiol..

[4] Palmer TD, Takahashi J, Gage FH (1997). The adult rat hippocampus contains primordial neural stem cells. Mol. Cell Neurosci..

[5] Imayoshi I (2008). Roles of continuous neurogenesis in the structural and functional integrity of the adult forebrain. Nat. Neurosci..

[6] Deng W, Aimone JB, Gage FH (2010). New neurons and new memories: how does adult hippocampal neurogenesis affect learning and memory?. Nat. Rev. Neurosci..

[7] Dupret D (2007). Spatial learning depends on both the addition and removal of new hippocampal neurons. Plos Biol..

[8] Sahay A (2011). Increasing adult hippocampal neurogenesis is sufficient to improve pattern separation. Nature.

[9] Saxe MD (2006). Ablation of hippocampal neurogenesis impairs contextual fear conditioning and synaptic plasticity in the dentate gyrus. Proc. Natl. Acad. Sci. USA.

[10] Barco A, Alarcon JM, Kandel ER (2002). Expression of constitutively active CREB protein facilitates the late phase of long-term potentiation by enhancing synaptic capture. Cell.

[11] Marie H, Morishita W, Yu X, Calakos N, Malenka RC (2005). Generation of silent synapses by acute *in vivo* expression of CaMKIV and CREB. Neuron.

[12] Merz K, Herold S, Lie DC (2011). CREB in adult neurogenesis–master and partner in the development of adult-born neurons?. Eur. J. Neurosci..

[13] Mizuno M (2002). CREB phosphorylation as a molecular marker of memory processing in the hippocampus for spatial learning. Behav. Brain Res..

[14] Silva AJ, Kogan JH, Frankland PW, Kida S (1998). CREB and memory. Annu. Rev. Neurosci..

[15] Suzuki A (2011). Upregulation of CREB-mediated transcription enhances both short- and long-term memory. J. Neurosci..

[16] Parker D (1996). Phosphorylation of CREB at Ser-133 induces complex formation with CREB-binding protein via a direct mechanism. Mol. Cell Biol..

[17] Shih HM (1996). A positive genetic selection for disrupting protein–protein interactions: Identification of CREB mutations that prevent association with the coactivator CBP. Proc. Natl. Acad. Sci. USA.

[18] Gouveia A (2016). The aPKC-CBP pathway regulates adult hippocampal neurogenesis in an age-dependent manner. Stem Cell Reports.

[19] He L (2012). Transcriptional Co-activator p300 Maintains Basal Hepatic Gluconeogenesis. J. Biol. Chem..

[20] He L (2009). Metformin and insulin suppress hepatic gluconeogenesis through phosphorylation of CREB binding protein. Cell.

[21] Chen G, Zou X, Watanabe H, van Deursen JM, Shen J (2010). CREB binding protein is required for both short-term and long-term Memory Formation. J. Neurosci..

[22] Oliverira AMM (2011). Subregion-specific p300 conditional knock-out mice exhibit long-term memory impairments. Learn. Mem..

[23] Peixoto L, Abel T (2013). The role of histone acetylation in memory formation and cognitive impairments. Neuropsychopharm. Reviews.

[24] Korzus E, Rosenfeld MG, Mayford M (2004). CBP Histone acetyltransferase activity is a critical component of memory consolidation. Neuron.

[25] Wood MA, Attner MA, Oliveira AMM, Brindle PK, Abel TA (2006). transcription factor-binding domain of the coactivator CBP is essential for long-term memory and the expression of specific target genes. Learn. Mem..

[26] Hawk JD (2012). NR4A nuclear receptors support memory enhancement by histone deacetylase inhibitors. J. Clin. Invest..

[27] Vecsey CG (2007). Histone deacetylase inhibitors enhance memory and synaptic plasticity via CREB: CBP-Dependent transcriptional activation. J. Neurosci..

[28] Matus-Amat P, Higgins EA, Barrientos RM, Rudy JW (2004). The role of the dorsal hippocampus in the acquisition and retrieval of context memory representations. J. Neurosci..

[29] Rudy JW, Huff NC, Matus-Amat P (2004). Understanding contextual fear conditioning: insights from a two-process model. Neurosci. Biobehav. Rev..

[30] Reed SM, Quelle DE (2015). p53 Acetylation: Regulation and Consequences. Cancers.

[31] Eisch AJ, Barrot M, Schad CA, Self DW, Nestler EJ (2000). Opiates inhibit neurogenesis in the adult rat hippocampus. Proc. Natl. Acad. Sci. USA.

[32] Gould E, Beylin A, Tanapat P, Reeves A, Shors TJ (1999). Learning enhances adult neurogenesis in the hippocampal formation. Nat. Neurosci..

[33] Malberg JE, Eisch AJ, Nestler EJ, Duman RS (2000). Chronic antidepressant treatment increases neurogenesis in adult rat hippocampus. J. Neurosci..

[34] Olariu A, Cleaver KM, Cameron HA (2007). Decreased neurogenesis in aged rats results from loss of granule cell precursors without lengthening of the cell cycle. J. Comp. Neurol..

[35] Wang J (2012). Metformin activates atypical PKC-CBP pathway to promote neurogenesis and enhance spatial memory formation. Cell Stem Cell.

[36] West MJ, Gundersen HJ (1990). Unbiased stereological estimation of the number of neurons in the human hippocampus. J. Comp. Neurol..

[37] Jayatissa MN, Henningsen K, West MJ, Wiborg O (2009). Decreased cell proliferation in the dentate gyrus does not associate with development of anhedonic-like symptoms in rats. Brain Res..

[38] Mouton, P. Principles and Practices of Unbiased Stereology: An Introduction for Bioscientists (The Johns Hopkins University Press, Baltimore) (2002).

[39] Guillery RW, Herrup K (1997). Quantification without pontification: choosing a method for counting objects in sectioned tissues. J. Comp. Neurol..

[40] Pakkenberg B, Gundersen HJ (1988). Total number of neurons and glial cells in human brain nuclei estimated by the disector and the fractionator. J. Microsc..

[41] West MJ, Slomianka L, Gundersen HJ (1991). Unbiased stereological estimation of the total number of neurons in thesubdivisions of the rat hippocampus using the optical fractionator. Anat. Rec..

[42] Carlo CN, Stevens CF (2011). Analysis of differential shrinkage in frozen brain sections and its implications for the use of guard zones in stereology. J. Comp. Neurol..

[43] Gardella D, Hatton WJ, Rind HB, Rosen GD, von Bartheld CS (2003). Differential tissue shrinkage and compression in the z-axis: implications for optical dissector counting in vibratome-, plastic- and cryosections. J. Neurosci. Methods.

[44] Miller DJ, Balaram P, Young NA, Kaas JH (2014). Three counting methods agree on cell and neuron number in chimpanzee primary visual cortex. Front. Neuroanat..

