# Supplementary material for: Ectopic expression of aPKC-mediated phosphorylation in p300 modulates hippocampal neurogenesis, CREB binding and fear memory differently with age
Source: Sci Rep. 2018 Sep 10;8:13489. doi: 10.1038/s41598-018-31657-2 (PMC6131509; doi:10.1038/s41598-018-31657-2)
Supplement: Supplementary file 1 — Supplementary figures [file 41598_2018_31657_MOESM1_ESM.docx]

**Ectopic expression of aPKC-mediated phosphorylation in p300 modulates hippocampal neurogenesis, CREB binding and fear memory differently with age**

Charvi Syal#^1,2^ , Matthew Seegobin#^1^, Sailendra Nath Sarma^1^, Ayden Gouveia^1,2^, , Karolynn Hsu^1^, Yosuke Niibori^4^, Ling He^5^, Fredric E.Wondisford^6^, Paul W. Frankland^4,7,8^, *Jing Wang^1,2,3^

^1^Regenerative Medicine Program, Ottawa Hospital Research Institute, Ottawa, Canada K1H 8L6; ^2^Department of Cellular and Molecular Medicine, ^3^Brain and Mind Research Institute, University of Ottawa, Ottawa, K1H 8M5, ^4^Neurosciences and Mental Health, Hospital for Sick Children, Toronto, ON M5G 1X8, Canada, ^95^Department of Pediatrics and Medicine, Johns Hopkins Medical School, Baltimore, MD 21287, USA; ^6^Department of Medicine, Rutgers-Robert Wood Johnson Medical School, New Brunswick, NJ 08901, USA.

Departments of ^7^Psychology, and ^8^Physiology, University of Toronto, Toronto, Canada M5G 1X5.

***Correspondence to**: Jing Wang, Regenerative Medicine Program, Ottawa Hospital Research Institute, Ottawa, Canada K1H 8L6. Email: jiwang@ohri.ca Telephone: 613-737-8899 ext. 71954.

#: Equal contribution


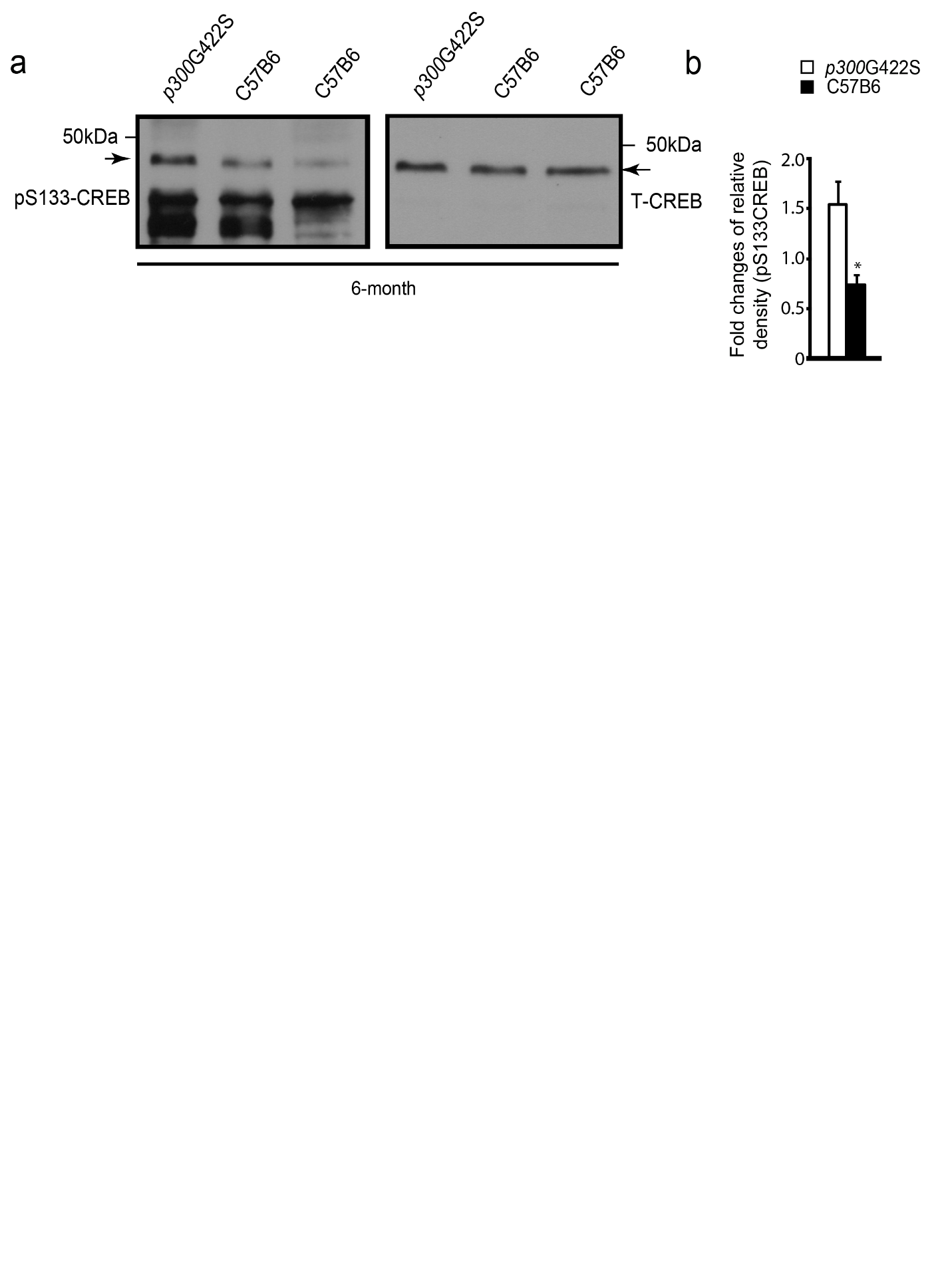
**Supplementary figure 1. C57B6 strain shows reduced pS133-CREB when compared to *p300*G422S strain at the age of 6 months hippocampi.** (a) Western blot analysis for pS133-CREB in hippocampal extracts from 6 months *p300*G422S strain and C57B6 strain. Blots were reprobed for total CREB as a loading control. (b) Graph shows relative levels of pS133-CREB over total CREB, normalized to one of 6 months *p300*G422S strain WT samples. *p < 0.05, n =3 animals for each group.

**
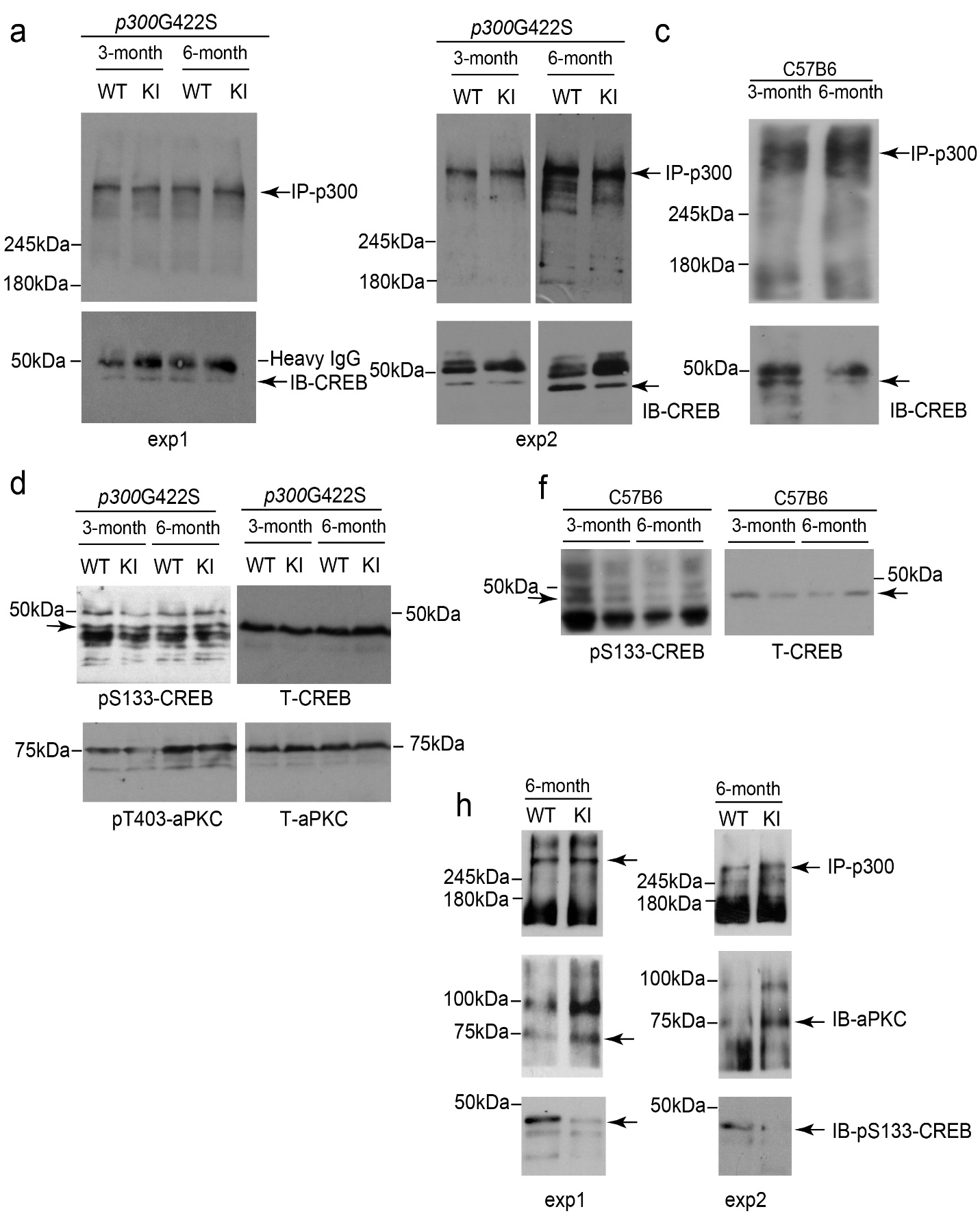
Supplementary figure 2:** Original gels from western blots in Figure 2.
